# Avoiding disentanglement of multipartite entangled optical beams with a correlated noisy channel

**DOI:** 10.1038/srep44475

**Published:** 2017-03-15

**Authors:** Xiaowei Deng, Caixing Tian, Xiaolong Su, Changde Xie

**Affiliations:** 1 State Key Laboratory of Quantum Optics and Quantum Optics Devices, Institute of Opto-Electronics, Shanxi University, Taiyuan 030006, China; 2 Collaborative Innovation Center of Extreme Optics, Shanxi University, Taiyuan 030006, China

## Abstract

A quantum communication network can be constructed by distributing a multipartite entangled state to space-separated nodes. Entangled optical beams with highest flying speed and measurable brightness can be used as carriers to convey information in quantum communication networks. Losses and noises existing in real communication channels will reduce or even totally destroy entanglement. The phenomenon of disentanglement will result in the complete failure of quantum communication. Here, we present the experimental demonstrations on the disentanglement and the entanglement revival of tripartite entangled optical beams used in a quantum network. We experimentally demonstrate that symmetric tripartite entangled optical beams are robust in pure lossy but noiseless channels. In a noisy channel, the excess noise will lead to the disentanglement and the destroyed entanglement can be revived by the use of a correlated noisy channel (non-Markovian environment). The presented results provide useful technical references for establishing quantum networks.

Quantum entanglement is a fundamental resource in quantum information tasks^1^. Considerable progress has been made in quantum information processing with entangled optical beams because the manipulation and measurement of the quadrature amplitudes of optical field are familiar in classical communication and processing technologies^2^,^3^,^4^. The used quantum variables, amplitude and phase quadratures, are just the analogies of position and momentum of a particle^2^. The multipartite entangled state can be used to complete one-way quantum computation^5^,^6^,^7^,^8^ and to construct quantum communication networks, such as quantum teleportation network^9^,^10^, controlled dense coding quantum communication^11^,^12^, and wavelength-multiplexed quantum network with ultrafast frequency comb^13^. Up to now, large scale multipartite entangled state with continuous variables has been experimentally prepared^14^,^15^,^16^, which provide necessary resource for quantum computation and quantum communication network.

In quantum communication networks, quantum states carrying information are transmitted between space-separated nodes through quantum channels, while losses and noises in channels will unavoidably lead to decoherence of quantum states. Decoherence, which is often caused by the interaction between system and the environment, is a main factor limiting the development of the quantum information technology. In quantum communication, the distributed entanglement will decrease because of the unavoidable decoherence in the quantum channel. In this case, entanglement purification, which is a way to distill highly entangled states from less entangled ones, is a necessary step to overcome decoherence^17^,^18^,^19^,^20^. Furthermore, it has been shown that decoherence will lead to entanglement sudden death (ESD)^21^,^22^, where two entangled qubits become completely disentangled in a finite-time under the influence of vacuum. ESD for multipartite entangled states has also been discussed theoretically^23^,^24^. Various methods to recover the bipartite entanglement after ESD occurred have been proposed and demonstrated during past several years, such as the non-Markovian environment^25^, weak measurement^26^, feedback^27^, *et al*. The fidelity of quantum teleportation directly depends on the entanglement degree of utilized quantum resource. If disentanglement occurs in a quantum teleportation network, the fidelity will never exceed its classical limit and thus the quantum communication will fail. Thus, it is necessary to investigate the physical conditions of reducing and destroying multipartite entanglement in quantum channels and explore the feasible schemes of avoiding disentanglement.

There are two types of quantum channels, the lossy channel and the noisy channel. In a lossy but noiseless (without excess noise) quantum channel, the noise induced by loss is nothing but the vacuum noise (corresponding to a zero-temperature environment)^4^. In a noisy channel, the excess noise higher than the vacuum noise exists^4^. Generally, the effect of loss and noise in quantum channel on the quantum state can be described by a quantum-noise-limited amplifier^28^,^29^,^30^,^31^. Since the effect of loss and excess noise in quantum channel on entangled optical beams are different^32^,^33^,^34^, we analyze the entanglement of a tripartite entangled optical beams over a lossy and noisy channel separately. It has been shown that the bipartite Gaussian entangled optical beams can be robust against loss^32^,^33^, while the three-color entanglement among three asymmetric optical modes can be fragile against loss due to the phonon noise in the generation system^35^. We extend the discussion of the robustness of entangled state over lossy channels to tripartite entangled optical beams, and experimentally demonstrate that the symmetric tripartite entangled state is robust against loss in quantum channels.

The excess noise in a communication channel is another main factor limiting the transmission of information, for example, it will decrease the secure transmission distance of quantum key distribution^4^. The noises in today’s communication systems exhibit correlations in time and space, thus it will be relevant to consider channels with correlated noise (non-Markovian environment)^36^,^37^,^38^. A correlated noisy channel has been used to complete Gaussian error correction^38^ and to protect squeezing in quantum communication with squeezed state over a noisy channel^39^. It has also been experimentally demonstrated that correlated noisy channel can be established by bundling two fibers together^40^. We study the entanglement property of the tripartite entangled optical beams over a noisy quantum channel, in which the disentanglement is observed. By applying an ancillary optical beam and establishing a correlated noisy channel, we successfully avoid disentanglement among the tripartite disentangled optical beams.

## Results

### Experimental scheme

The quantum state used in the experiment is a continuous variable Greenberger-Horne-Zeilinger (GHZ) tripartite entangled state^10^,^11^, which is prepared deterministically. The correlation variances between the amplitude (position) and phase (momentum) quadratures of the tripartite entangled state are expressed by 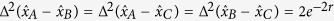 and 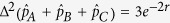, respectively, where the subscripts correspond to different optical modes (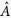, 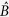 and 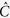) and *r* is the squeezing parameter (*r* = 0 and *r* = +∞ correspond to no squeezing and the ideally perfect squeezing, respectively). Obviously, in the ideal case with infinite squeezing (*r* → ∞), these correlation variances will vanish and the better the squeezing, the smaller the noise terms.

**Figure 1:**
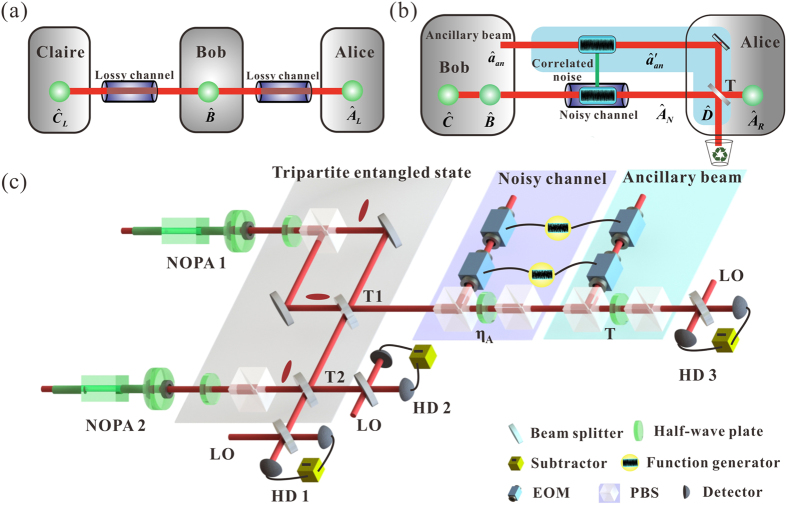
Schematic of principle and experimental set-up. (**a**) Two modes (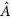 and 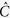) of a tripartite entangled state are distributed over two lossy quantum channels to Alice and Claire, respectively. (**b**) One mode of the tripartite entangled state is distributed over a noisy channel, where disentanglement is observed among optical modes 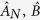 and 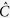. The entanglement revival operation is implemented by coupling an ancillary beam (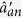) who has correlated noise with the environment with the transmitted mode 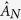 on a beam-splitter with transmission efficiency of 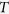, thus the entanglement among modes 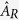, 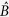 and 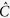 is revived. (**c**) The schematic of experimental set-up for distributing a mode of the tripartite entangled optical beams over a noisy channel and the entanglement revival. T1 and T2 are the beam-splitters used to generate the GHZ entangled state. *η*_*A*_ and *T* are the transmission efficiencies of the noisy channel and the revival beam-splitter, respectively. HD1-3, homodyne detectors. LO, the local oscillator.

When two optical modes (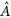 and 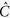) of a tripartite entangled state are distributed by Bob (who retains mode 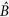) to two nodes (Alice and Claire) over two lossy channels, a quantum network with three users Alice, Bob and Claire is established [Fig. 1(a)]. After the transmission of optical modes 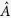 and 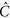 over two lossy channels, the output modes are given by 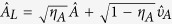 and 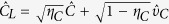, where *η*_*A(C*)_ and 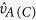 represent the transmission efficiency of the quantum channel and the vacuum state induced by loss into the quantum channel, respectively. When *η*_*A*_ ≠ 1, *η*_*C*_ = 1, it corresponds to the situation that the optical mode 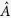 is distributed over a lossy channel to another node while modes 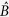 and 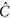 are maintained in a node. When mode 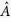 is distributed over a noisy channel [Fig. 1(b)], the transmitted mode is expressed by


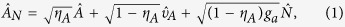


where 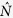 and *g*_*a*_ represent the Gaussian noise in the channel and the magnitude of noise, respectively. The excess noise on the transmitted mode will possibly lead to the disentanglement of the tripartite entangled state. In order to avoid disentanglement of the tripartite entangled state, an ancillary beam with correlated noise and a revival beam-splitter with the transmission coefficient *T* are used. The ancillary beam carrying correlated noise is expressed by 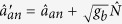, where 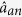 is the ancillary beam and *g*_*b*_ describes the magnitude of the correlated noise, which is an adjustable parameter in experiments. The transmitted 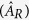 and reflected 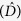 beams from the revival beam-splitter are 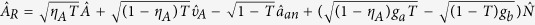 and 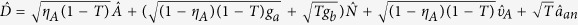, respectively. If the values of *g*_*b*_ and *T* are chosen to satisfy the following expression


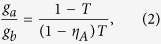


the noise on the output mode 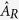 will be removed totally. In this case, the output mode becomes


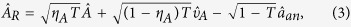


which is immune from the excess noise. The excess noise is transferred onto the reflected beam 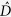 (which is abandoned) due to the use of the beam-splitter. Thus the tripartite entanglement among 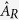, 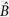 and 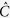 is preserved by using a correlated noisy channel.

### Experimental set-up

The experimental set-up for distributing a mode of the tripartite entangled optical beams over a noisy channel and the entanglement revival is shown in Fig. 1(c). The nondegenerate optical parametric amplifiers (NOPAs) are pumped by a common laser source, which is a continuous wave intracavity frequency-doubled and frequency-stabilized Nd:YAP-LBO (Nd-doped YAlO_3_ perorskite-lithium triborate) laser. Each of the NOPAs consists of an *α*-cut type-II KTP crystal and a concave mirror. The front face of the KTP crystal was coated to be used for the input coupler and the concave mirror serves as the output coupler of the squeezed states. The transmissions of the input coupler at 540 nm and 1080 nm are 99.8% and 0.04%, respectively. The transmissions of the output coupler at 540 nm and 1080 nm are 0.5% and 5.2%, respectively. A pair of 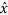-squeezed and 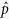-squeezed states in two orthogonal polarizations are produced by NOPA1^41^. The other 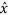-squeezed state is produced by NOPA2. NOPAs are locked individually by using Pound-Drever-Hall method with a phase modulation of 56 MHz on 1080 nm laser beam^42^. Both NOPAs are operated at deamplification condition, which corresponds to lock the relative phase between the pump laser and the injected signal to (2n + 1)*π* (n is the integer).

The tripartite entangled state of optical field at the sideband frequency of 2 MHz is obtained by combining three squeezed states of light with −3.5 dB squeezing and 8.5 dB anti-squeezing on two optical beam-splitters with transmission coefficients *T*_1_ = 1/3 and *T*_2_ = 1/2, respectively (see Supplementary Information for details). For a real communication channel, the loss and noise are coming from the environment as that shown in Fig. 1. The loss in the quantum channel is mimicked by a beam-splitter composed by a half-wave plate and a polarization beam-splitters (PBS). The noisy channel is simulated by adding a Gaussian noise on a coherent beam with electro-optic modulators (EOMs) and then the modulated beam is coupled with the transmitted mode on a beam-splitter with transmission efficiency *η*_*A*_. When an ancillary beam with the correlated noise is mixed with the transmitted mode on a beam-splitter of the transmission coefficient *T*, the entanglement revival is completed. The covariance matrix of the output state is measured by three homodyne detectors. The quantum efficiency of the photodiodes used in the homodyne detectors are 95%. The interference efficiency on all beam-splitters are about 99%.

### The positive partial transposition criterion

The positive partial transposition (PPT) criterion^43^,^44^ is a necessary and sufficient condition for judging the existence of quantum entanglement among *N* Gaussian optical beams, when the state has the form of bipartite splitting with only a single mode on one side like (1|*N* − 1)^45^,^46^. We use the PPT criterion to verify the disentanglement and the entanglement revival of the tripartite entangled states of light. Based on above-mentioned expressions of output state, we obtain the covariance matrix, which is given in the Supplementary Information, and calculate the symplectic eigenvalues. The positivity is checked by evaluating the symplectic eigenvalues of the partially transposed matrix and the state is separable if any of the symplectic eigenvalues is larger than or equal to 1^47^.

At the level of quadrature operators, the partial transposition with respect to mode *k (k* = 1, 2, 3) corresponds to the change of sign of the phase quadrature, i. e. 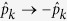. Symplectic eigenvalues of covariance matrix are defined as the positive roots of the polynomial 
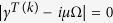
, where 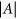 denotes the determinant of matrix^47^. 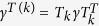 is the partially transposed matrix of the quantum state, where *T*_*k*_ is a diagonal matrix with all diagonal elements equal to 1 except for *T*_2*k*,2*k*_ = −1, and


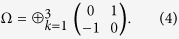


We consider a bipartite splitting of a three-mode Gaussian state with covariance matrix *γ* such that one party holds mode *k* and the other party possesses the remained two modes. If the smallest symplectic eigenvalue *μ*_*k*_ obtained from the polynomial is smaller than 1, the state is inseparable with respect to the *k*|*ij* splitting.

### Entanglement in lossy channels

**Figure 2:**
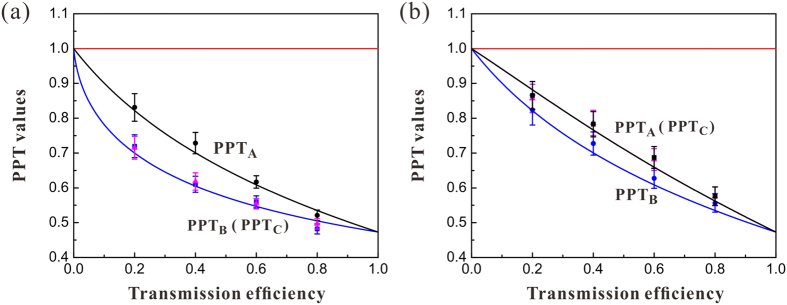
The entanglement in lossy channels. (**a**) One optical mode 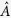 is distributed in the lossy channel (*η*_*A*_ ≠ 1, *η*_*C*_ = 1). (**b**) Two optical modes 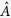 and 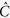 are distributed in the lossy channels with the same transmission efficiency (*η*_*A*_ ≠ 1, *η*_*C*_ ≠ 1). PPT values are all below the entanglement boundary (red lines), which means that the tripartite entanglement is robust against loss in quantum channels. The black, blue and pink dots represent the experimental data for different PPT values, respectively. Error bars represent ± one standard deviation and are obtained based on the statistics of the measured noise variances.

Figure 2 shows that the tripartite entanglement is robust against loss in lossy quantum channels. The PPT values PPT_A_, PPT_B_ and PPT_C_ represent the different splittings for the (A|BC), (B|AC) and (C|AB), respectively. The PPT values of distributing one optical mode 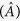 and two optical modes (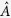 and 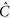) in one and two lossy channels (for simplification, we assume the losses in two quantum channels are the same) are shown in Fig. 2(a) and (b), respectively. The values of PPT_B_ and PPT_C_ in Fig. 2(a) [PPT_A_ and PPT_C_ in Fig. 2(b)] are the same because that the optical modes of the tripartite entangled state are symmetric and modes 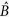 and 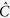 do not interact with the environment (modes 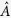 and 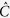 are transmitted with the same transmission efficiency). Comparing the corresponding PPT values in Fig. 2(a) and (b), we can see that the tripartite entanglement is more robust if only a mode 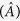 pass through the lossy channel than that both modes 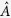 and 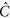 subject to the lossy channels, i. e. the degradation of entanglement in the case of *η*_*C*_ = 1 is less than that in the case of *η*_*A*_ ≠ 1 and *η*_*C*_ ≠ 1. In lossy channels, the tripartite entanglement gradually decreases along with the degradation of the transmission efficiency of quantum channel and finally tends to zero when the channel efficiency equals to zero. This is different from the results in ref. 35, where the disentanglement of a tripartite entangled light beam is observed over a lossy channel. We theoretically analyze the physical reason of the difference based on the covariance matrix and show that it is because the tripartite entangled state prepared by us is a symmetric entangled state, while the state in ref. 35 is an asymmetric state since the effect of the classical phonon noise in the optical parametric oscillator^48^, which are discussed detailedly at next section.

### Discussion on symmetric and asymmetric states

We consider the case of the transmission in two lossy channels for tripartite Gaussian symmetric and asymmetric optical states, respectively. For convenience, the covariance matrix of the original tripartite Gaussian state is written in terms of two-by-two submatrices as


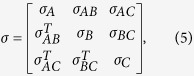


in which each diagonal block *σ*_*k*_ is the local covariance matrix corresponding to the reduced state of mode *k (k* = *A, B, C*), respectively, and the off-diagonal matrices *σ*_*mn*_ are the intermodal correlations between subsystems *m* and *n*. The detailed expressions of *σ*_*k*_ and *σ*_*mn*_ are given in the Supplementary Information.

#### Type I symmetric state

If a quantum state has symmetric modes (*σ*_*A*_ = *σ*_*B*_ = *σ*_*C*_) and balanced correlations between subsystems *σ*_*mn*_, i. e. the absolute values of main diagonal elements in *σ*_*mn*_ are the same, we say it is a symmetric state, where the variances are 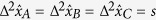, 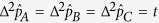, 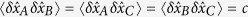, 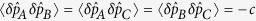, and 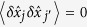. The corresponding covariance matrix is


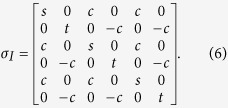


This type of quantum state can be generated by the interference of three squeezed states on beam-splitter network. The three squeezed states are produced from three NOPAs operating below its oscillation threshold, respectively^41^. The experimental values of parameters c, s and t in Eq. (6) are obtained by the covariance matrix of the entangled state prepared by us [see Eq. (6) in the Supplementary Information]. Apparently, the prepared state is a symmetric state. According to the theoretical calculation result shown in Fig. 2, in which all used parameters in the calculation are derived from Eqs. (6) and (7) in the Supplementary Information, it is proved that the symmetric quantum states are fully robust against losses in the two lossy channels.

#### Type II asymmetric state

The asymmetric quantum state has unbalanced correlations between subsystems (*c*_*x*_ ≠ *c*), whose covariance matrix is given by


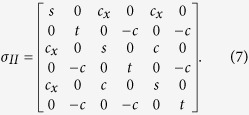


**Figure 3:**
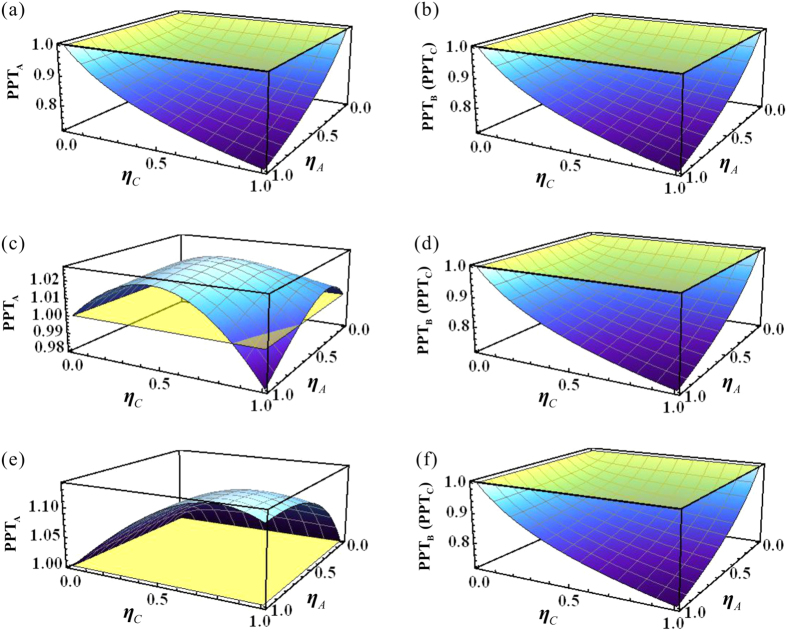
The entanglement properties of different asymmetric tripartite states in two lossy channels. (**a**,**b**) PPT values of fully robust entanglement against loss with *c*_*x*_/*c* = 0.8. (**c**,**d**) PPT values of a one-mode fragile state in lossy channels for attenuations with *c*_*x*_/*c* = 0.5. (**e**,**f**) A totally one-mode biseparable state in lossy channels with *c*_*x*_/*c* = 0.3.

The entanglement property of the asymmetric state is shown in Fig. 3 [all parameters used in the calculation are also taken from Eqs (6) and (7) in the Supplementary Information]. The mathematic operation of modifying a parameter, *c*_*x*_, and keeping other elements in the covariance matrix unchanging is equivalent to physically add an uncorrelated noise into the generation system of the tripartite entangled state^33^. For example, the phonon noise in the prepared three-color entangled state in ref. 35, which is produced by a non-degenerated optical parametric oscillator operating above its oscillation threshold, is a type of classical and uncorrelated noises. In an above-threshold NOPO, the effect of the uncorrelated phonon noise is large and thus the produced three-color entangled state is asymmetric^35^. In Eq. (7), we assume that the different amounts of the uncorrelated noise exist in mode 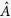, thus *c*_*x*_ in both *σ*_*AB*_ and *σ*_*AC*_ is changed simultaneously, while *σ*_*BC*_ is unchanged because it is not related to mode 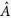. Figure 3(a) and (b) are the PPT values corresponding to *c*_*x*_/*c* = 0.8, which show that the disentanglement never happens, thus the original state is robust against losses. Actually, all asymmetric states correspond to *c*_*x*_/*c* > 0.8 are robust against losses according to our calculation. Figure 3(c) and (d) are the PPT values corresponding to *c*_*x*_/*c* = 0.5, which show that the disentanglement happens for A|BC during decreasing the transmission efficiencies, thus the original state is an one-mode fragile state for the attenuations. From Fig. 3(e) and (f) we can see that the state is a totally one-mode biseparable state when *c*_*x*_/*c* = 0.3. Thus the original asymmetric tripartite state evolutes from a robust state to a fully one-mode biseparable state with the decrease of the *c*_*x*_ value. We should emphasize that the asymmetric property of quantum states does not certainly result in the disentanglement, and only when the uncorrelated noise in the quantum state is large enough the disentanglement occurs. To the physical reason, the correlation among subsystems in a symmetric state is balanced, i. e. there is no uncorrelated noise in the quantum state. Thus all entangled modes for the symmetric state are equivalent and the entanglement is reduced gradually and continually in a lossy channel. However, for an asymmetric state, the correlation among subsystems is unbalanced, i. e. uncorrelated noises are added into the quantum state, which lead to disentanglement of the asymmetric tripartite state.

### Entanglement in noisy channel

**Figure 4:**
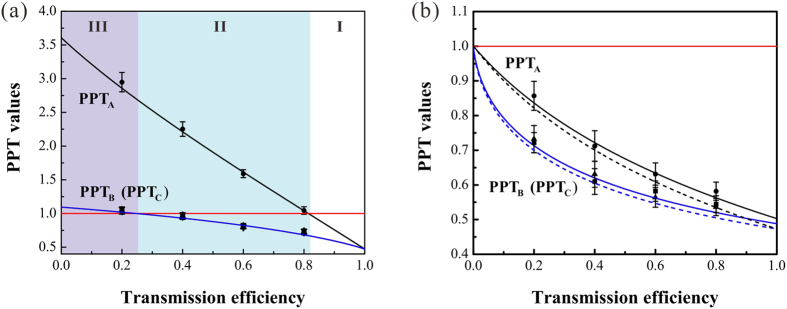
The disentanglement and entanglement revival in a noisy channel. (**a**) The PPT values for the transmission in a noisy channel, where the variance of the excess noise is taken as five times of shot noise level. The tripartite entangled state experiences entanglement (I), one-mode biseparable (II) and fully disentanglement (III) along with the decreasing of the channel efficiency. (**b**) The PPT values after entanglement revival. Dash lines are the corresponding results of the perfectly revival, the results are the same with the lines in Fig. 2(a) which are obtained before disentanglement. The black dots represent the experimental data. Error bars represent ± one standard deviation and are obtained based on the statistics of the measured noise variances.

When there is the excess noise in the quantum channel, the disentanglement is observed as shown in Fig. 4, where the variance of the excess noise is taken as five times of shot noise level and *g*_*a*_ = 1. The values of PPT_B_ and PPT_C_ are the same because the tripartite entangled state is symmetric and both modes 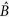 and 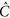 are retained in a node. In this case, we can assume that modes 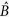 and 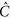 have no interaction with the environment. Entanglement survives when the transmission efficiency satisfies 0.81 < *η* ≤ 1 [region I in Fig. 4(a)]. When 0.25 < *η* ≤ 0.81 PPT_A_ is above 1 while PPT_B_ and PPT_C_ are below 1, the state is corresponding to a one-mode biseparable state [region II in Fig. 4(a)], which means that mode 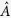 is separated from modes 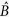 and 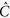. When the transmission efficiency is *η* ≤ 0.24, fully disentanglement is observed [region III in Fig. 4(a)], which will result in that the quantum communication between any two users is not possible to be implemented.

There are two adjustable parameters in the entanglement revival procedure, *g*_*b*_ and *T*. For different channel efficiency, we may fix one of them and adjust the other one to recover the entanglement according to Eq. (2). In the experiment, we chose to fix *T* and adjust *g*_*b*_ for different transmission efficiencies of the quantum channel. Generally, *T* can not be taken too small because it corresponds to add a linear loss on the transmitted mode, which will degrade the tripartite entanglement. The solid and dash lines in Fig. 4(b) represent the revived entanglement in our experiment with *T* = 90% and in the ideal case with the perfectly revival, respectively. The imperfect transmission efficiency of the revival beam-splitter leads to the small difference between the two results. The parameters *g*_*a*_/*g*_*b*_ are chosen to be 0.14, 0.18, 0.28 and 0.56 for channel efficiencies of 0.2, 0.4, 0.6 and 0.8, respectively. Figure 4(b) shows that the entanglement is revived after the revival operation.

**Figure 5:**
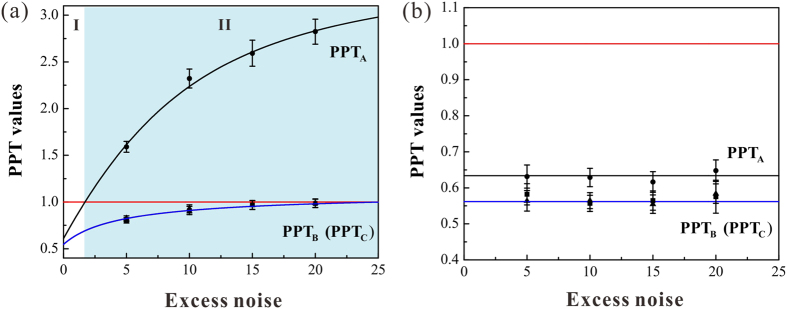
The disentanglement and revival of entanglement at different noise levels (in the unit of shot noise level). (**a**) Disentanglement at different excess noise levels. (**b**) The PPT values after entanglement revival, and it is independent on the excess noise. The black dots represent the experimental data. Error bars represent ± one standard deviation and are obtained based on the statistics of the measured noise variances.

The dependence of entanglement on the excess noise is shown in Fig. 5, where the transmission efficiency is chosen to be 0.6, *g*_*a*_/*g*_*b*_ = 0.28, and *T* = 90%. We can see that the boundary of disentanglement depends on the excess noise level. When the variance of the excess noise is lower than 2.2 times of shot noise level for the transmission efficiency of *η* = 0.6, the entanglement can be survived in a noisy channel. When the variance of the excess noise is higher than 2.2 times of shot noise level for the transmission efficiency *η* = 0.6, the disentanglement happens and the state is reduced to an one-mode biseparable state [region II in Fig. 5(a)]. After the revival operation, the entanglement is recovered and it is independent on the excess noise as that indicated by Eq. (3).

## Discussion

In summary, we investigate the different effects of the lossy channel and the noisy channel on the tripartite entangled state of light when it is distributed in a quantum network and demonstrate that the entangled optical beams with the symmetric structure is more robust to the channel losses than that with the asymmetric structure. For the asymmetric tripartite entangled state of light, the robustness of the state in lossy channel depends on the correlation between subsystems. Disentanglement is observed when the excess noise exists in the quantum channel. By creating a correlated noisy channel (non-Markovian environment), entanglement of the tripartite entangled state is preserved, thus disentanglement can be avoided with the correlated noisy channel. But when the revived state passed through a noisy channel again the disentanglement will possibly appear again.

The correlated noisy channel used in our experiment can only remove the effect of excess noise in the quantum channel, while the influence of loss can not be eliminated by the scheme. Fortunately, another technology, the noiseless linear amplification, can be used to eliminate the effect of loss on entanglement^49^,^50^,^51^,^52^. Due to that the disentanglement induced by environment is a specific phenomenon among correlated quantum systems, which is never observed in the studies of dissipation effects for classical systems, the presented results are significant to understand the dynamic behavior of the interaction of entangled states and different environment. Besides, our investigation also provides concrete references for establishing quantum network with multipartite entangled states of light.

## Additional Information

**How to cite this article**: Deng, X. *et al*. Avoiding disentanglement of multipartite entangled optical beams with a correlated noisy channel. *Sci. Rep.* **7**, 44475; doi: 10.1038/srep44475 (2017).

**Publisher's note:** Springer Nature remains neutral with regard to jurisdictional claims in published maps and institutional affiliations.

## Supplementary Material

Supplementary Information

## References

[b1] Nielson M. A. & Chuang I. L. Quantum Computation And Quantum Information. (Cambridge University, Cambridge, 2000).

[b2] Ralph T. C. & Lam P. K. A bright future for quantum communications. Nat. Photon. 3, 671–673 (2009).

[b3] Braunstein S. L. & van Loock P. Quantum information with continuous variables. Rev. Mod. Phys. 77, 513–577 (2005).

[b4] Weedbrook C. . Gaussian quantum information. Rev. Mod. Phys. 84, 621–669 (2012).

[b5] Raussendorf R. & Briegel H. J. A one-way quantum computer. Phys. Rev. Lett. 86, 5188–5191 (2001). 11384453 10.1103/PhysRevLett.86.5188

[b6] Walther P. . Experimental one-way quantum computing. Nature 434, 169–176 (2005). 15758991 10.1038/nature03347

[b7] Ukai R. . Demonstration of unconditional one-way quantum computations for continuous variables. Phys. Rev. Lett. 106, 240504 (2011). 21770557 10.1103/PhysRevLett.106.240504

[b8] Su X. . Gate sequence for continuous variable one-way quantum computation. Nat. Commun. 4, 2828 (2013).

[b9] van Loock P. & Braunstein S. L. Multipartite entanglement for contonuous variables: a quantum teleportation network. Phys. Rev. Lett. 84, 3482–3485 (2000). 11019120 10.1103/PhysRevLett.84.3482

[b10] Yonezawa H., Aoki T. & Furusawa A. Demonstration of a quantum teleportation network for continuous variables. Nature 431, 430–433 (2004). 15386006 10.1038/nature02858

[b11] Jing J. . Experimental demonstration of tripartite entanglement and controlled dense coding for continuous variables. Phys. Rev. Lett. 90, 167903 (2003). 12732011 10.1103/PhysRevLett.90.167903

[b12] Shen H., Su X., Jia X. & Xie C. Quantum communication network utilizing quadripartite entangled states of optical field. Phys. Rev. A 80, 042320 (2009).

[b13] Roslund J., de Araújo R. M., Jiang S., Fabre C. & Treps N. Wavelength-multiplexed quantum networks with ultrafast frequency combs. Nat. Photon. 8, 109–112 (2014).

[b14] Su X. . Experimental preparation of eight-partite cluster state for photonic qumodes. Opt. Lett. 37, 5178–5180 (2012). 23258044 10.1364/OL.37.005178

[b15] Yokoyama S. . Ultra-large-scale continuous-variable cluster states multiplexed in the time domain. Nature Photon. 7, 982–986 (2013).

[b16] Chen M. Menicucci N. C. & Pfister O. Experimental realization of multipartite entanglement of 60 modes of a quantum optical frequency comb. Phys. Rev. Lett. 112, 120505 (2014). 24724640 10.1103/PhysRevLett.112.120505

[b17] Pan J. W., Simon C., Brukner Č. & Zeilinger A. Entanglement purification for quantum communication. Nature 410, 1067 (2001). 11323664 10.1038/35074041

[b18] Duan L. M., Giedke G., Cirac J. I. & Zoller P. Entanglement purification of Gaussian continuous variable quantum states. Phys. Rev. Lett. 84, 4002 (2000). 11019260 10.1103/PhysRevLett.84.4002

[b19] Hage B. . Preparation of distilled and purified continuous-variable entangled states. Nat. Phys. 4, 915 (2008).

[b20] Dong R. . Experimental entanglement distillation of mesoscopic quantum states. Nat. Phys. 4, 919 (2008).

[b21] Yu T. & Eberly J. H. Sudden death of entanglement. Science 323, 598–601 (2009). 19179521 10.1126/science.1167343

[b22] Almeida M. P. . Environment-induced sudden death of entanglement. Science 316, 579–582 (2007). 17463284 10.1126/science.1139892

[b23] López C. E., Romero G., Lastra F., Solano E. & Retamal J. C. Sudden birth versus sudden death of entanglement in multipartite systems. Phys. Rev. Lett. 101, 080503 (2008). 18764597 10.1103/PhysRevLett.101.080503

[b24] Aolita L., Chaves R., Cavalcanti D., Acn A. & Davidovich L. Scaling laws for the decay of multiqubit entanglement. Phys. Rev. Lett. 100, 080501 (2008). 18352609 10.1103/PhysRevLett.100.080501

[b25] Xu J. S. . Experimental demonstration of photonic entanglement collapse and revival. Phys. Rev. Lett. 104, 100502 (2010). 20366406 10.1103/PhysRevLett.104.100502

[b26] Kim Y. S., Lee J. C., Kwon O. & Kim Y. H. Protecting entanglement from decoherence using weak measurement and quantum measurement reversal. Nat. Phys. 8, 117–120 (2012).

[b27] Yamamoto N., Nurdin H. I., James M. R. & Petersen I. R. Avoiding entanglement sudden death via measurement feedback control in a quantum network. Phys. Rev. A 78, 042339 (2008).

[b28] Haus H. A. & Mullen J. A. Quantum noise in linear amplifiers. Phys. Rev. 128, 2407 (1962).

[b29] Caves C. M. Quantum limits on noise in liear amplifiers. Phys. Rev. D 26, 1817 (1982).

[b30] Zavatta A., Fiurášek J. & Bellini M. A high-fidelity noiseless amplifier for quantum light states. Nature Photon. 5, 52–60 (2011).

[b31] Josse V., Sabuncu M., Cerf N. J., Leuchs G. & Andersen U. L. Universal optical amplification without nonlinearity. Phys. Rev. Lett. 96, 163602 (2006). 16712228 10.1103/PhysRevLett.96.163602

[b32] Barbosa F. A. S. . Robustness of bipartite Gaussian entangled beams propagating in lossy channels. Nat. Photon. 4, 858–861 (2010).

[b33] Barbosa F. A. S. . Disentanglement in bipartite continuous-variable systems. Phys. Rev. A 84, 052330 (2011).

[b34] Su X. Effect of excess noise on continuous variable entanglement sudden death and Gaussian quantum discord. Chin. Phys. B 22, 080304 (2013).

[b35] Coelho A. S. . Three-color entanglement. Science 326, 823–826 (2009). 19762598 10.1126/science.1178683

[b36] Kretschmann D. & Werner R. F. Quantum channels with memory. Phys. Rev. A 72, 062323 (2005).

[b37] Corney J. F. . Many-Body Quantum dynamics of polarization squeezing in optical fibers. Phys. Rev. Lett. 97, 023606 (2006). 16907445 10.1103/PhysRevLett.97.023606

[b38] Lassen M., Berni A., Madsen L. S., Filip R. & Andersen U. L. Gaussian error correction of quantum states in a correlated noisy channel. Phys. Rev. Lett. 111, 180502 (2013). 24237495 10.1103/PhysRevLett.111.180502

[b39] Deng X. . Disappearance and revival of squeezing in quantum communication with squeezed state over a noisy channel. Appl. Phys. Lett. 108, 081105 (2016).

[b40] Xu J. . Robust bidirectional links for photonic quantum networks. Sci. Adv. 2, e1500672 (2016). 26824069 10.1126/sciadv.1500672 PMC4730861

[b41] Zhang Y. . Experimental generation of bright two-mode quadrature squeezed light from a narrow-band nondegenerate optical parametric amplifier. Phys. Rev. A 62, 023813 (2000).

[b42] Drever R. W. P. . Laser phase and frequency stabilization using an optical resonator. Appl. Phys. B 31, 97 (1983).

[b43] Horodecki M., Horodecki P. & Horodecki R. Separability of mixed states: necessary and sufficient conditions. Phys. Lett. A 223, 1–8 (1996).

[b44] Simon R. Peres-Horodecki separability criterion for continuous variable systems. Phys. Rev. Lett. 84, 2726–2729 (2000). 11017310 10.1103/PhysRevLett.84.2726

[b45] Werner R. F. & Wolf M. M. Bound entangled Gaussian states. Phys. Rev. Lett. 86, 3658–3661 (2001). 11328047 10.1103/PhysRevLett.86.3658

[b46] Adesso G., Serafini A. & Illuminati F. Multipartite entanglement in three-mode Gaussian states of continuous-variable systems: Quantification, sharing structure, and decoherence. Phys. Rev. A 73, 032345 (2006).

[b47] Vollmer C. E. . Experimental entanglement distribution by separable states. Phys. Rev. Lett. 111, 230505 (2013). 24476242 10.1103/PhysRevLett.111.230505

[b48] Villar A. S., Martinelli M., Fabre C. & Nussenzveig P. Direct production of tripartite pump-signal-idler entanglement in the above-threshold optical parametric oscillator. Phys. Rev. Lett. 97, 140504 (2006). 17155232 10.1103/PhysRevLett.97.140504

[b49] Ralph T. C. Quantum error correction of continuous-variable states against gaussian noise. Phys. Rev. A 84, 022339 (2011).

[b50] Xiang G. Y., Ralph T. C., Lund A. P., Walk N. & Pryde G. J. Heralded noiseless linear amplification and distillation of entanglement. Nature Photon. 4, 316–319 (2010).

[b51] Chrzanowski H. M. . Measurement-based noiseless linear amplification for quantum communication. Nature Photon. 8, 333–338 (2014).

[b52] Ulanov A. E. . Undoing the effect of loss on quantum entanglement. Nature Photon. 9, 764–768 (2015).

