# Supplementary material for: Avoiding disentanglement of multipartite entangled optical beams with a correlated noisy channel
Source: Sci Rep. 2017 Mar 15;7:44475. doi: 10.1038/srep44475 (PMC5353719; doi:10.1038/srep44475)
Supplement: Supplementary Information [file srep44475-s1.pdf]

Supplementary Information for "Avoiding disentanglement of multipartite entangled optical beams with a correlated noisy channel"

Xiaowei Deng[1,2], Caixing Tian[1,2], Xiaolong Su[1,2],* and Changde Xie[1,2]
[1] State Key Laboratory of Quantum Optics and Quantum Optics Devices, Institute of Opto-Electronics, Shanxi University, Taiyuan, 030006, People's Republic of China.
[2] Collaborative Innovation Center of Extreme Optics, Shanxi University, Taiyuan,Shanxi 030006, People's Republic of China.

I. PREPARATION OF THE TRIPARTITE ENTANGLED STATE

The tripartite entangled state used in the experiment is a continuous variable tripartite Greenberger-Horne-Zeilinger (GHZ) state of optical field [1] which is prepared by coupling a phase-squeezed state ($\hat{a}_2$) of light and two amplitude-squeezed states of light ($\hat{a}_1$ and $\hat{a}_3$) on an optical beam-splitter network, which consists of two optical beam-splitters with transmittance of $T_1 = 1/3$ and $T_2 = 1/2$, respectively, as shown in Fig. 1. Three input squeezed states are expressed by

$$\begin{aligned}\hat{a}_1 &= e^{-r_1}\hat{x}_1^{(0)} + ie^{r_1}\hat{p}_1^{(0)},\\ \hat{a}_2 &= e^{r_2}\hat{x}_2^{(0)} + ie^{-r_2}\hat{p}_2^{(0)},\\ \hat{a}_3 &= e^{-r_3}\hat{x}_3^{(0)} + ie^{r_3}\hat{p}_3^{(0)},\end{aligned} \quad (1)$$

where r_i $(i = 1, 2, 3)$ is the squeezing parameter, $\hat{x} = \hat{a} + \hat{a}^\dagger$ and $\hat{p} = (\hat{a} - \hat{a}^\dagger)/i$ are the amplitude and phase quadratures of an optical field $\hat{a}$, respectively, and the superscript of the amplitude and phase quadratures represent the vacuum state. The transformation matrix of the beam-splitter network is given by

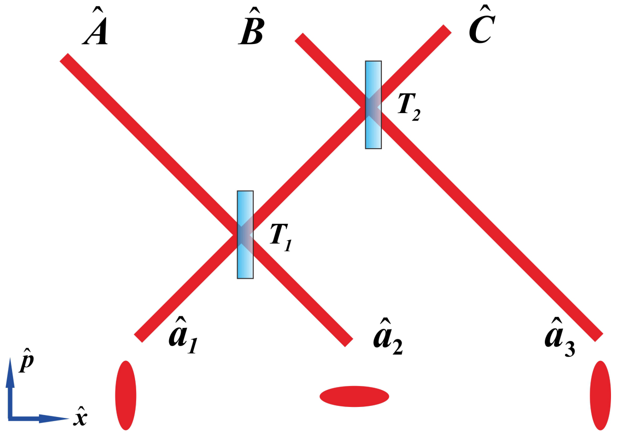

FIG. 1: The schematic of generation system of the tripartite entangled state.

$$U = \begin{bmatrix} \sqrt{\frac{2}{3}} & \sqrt{\frac{1}{3}} & 0 \\ -\sqrt{\frac{1}{6}} & \sqrt{\frac{1}{3}} & \sqrt{\frac{1}{2}} \\ -\sqrt{\frac{1}{6}} & \sqrt{\frac{1}{3}} & -\sqrt{\frac{1}{2}} \end{bmatrix}, \quad (2)$$

the unitary matrix can be decomposed into a beam-splitter network $U = B_{23}^{+}(T_2)I_2(-1)B_{12}^{+}(T_1)$, where $B_{kl}^{+}(T_j)$ stands for the linearly optical transformation on jth beam-splitter with transmission of T_j $(j = 1, 2)$, where $(B_{kl}^{+})_{kk} = \sqrt{1-T}, (B_{kl}^{+})_{kl} = (B_{kl}^{+})_{lk} = \sqrt{T}, (B_{kl}^{+})_{ll} = -\sqrt{1-T}$, are matrix elements of the beam-splitter. $I_k(-1) = e^{i\pi}$ corresponds to a 180° rotation in phase space. The output modes from the optical beam-splitter network are expressed by

$$\begin{aligned}\hat{A} &= \sqrt{\frac{2}{3}}\hat{a}_1 + \sqrt{\frac{1}{3}}\hat{a}_2,\\ \hat{B} &= -\sqrt{\frac{1}{6}}\hat{a}_1 + \sqrt{\frac{1}{3}}\hat{a}_2 + \sqrt{\frac{1}{2}}\hat{a}_3,\\ \hat{C} &= -\sqrt{\frac{1}{6}}\hat{a}_1 + \sqrt{\frac{1}{3}}\hat{a}_2 - \sqrt{\frac{1}{2}}\hat{a}_3,\end{aligned} \quad (3)$$

respectively. Here, we have assumed that three squeezed states have the identical squeezing parameter ($r_1 = r_2 = r_3$). In experiments, the requirement is easily achieved by adjusting the two nondegenerate optical parametric amplifiers (NOPAs) to operate at totally same conditions. For our experimental system, we have $r = 0.4$.

II. COVARIANCE MATRIX OF THE TRIPARTITE OPTICAL BEAMS

Gaussian state is the state with Gaussian characteristic functions and quasi-probability distributions on the multi-mode quantum phase space, which can be completely characterized by a covariance matrix. The elements of the tripartite covariance matrix are $\sigma_{ij} = Cov\left(\hat{R}_i, \hat{R}_j\right) = \frac{1}{2}\left\langle \hat{R}_i\hat{R}_j + \hat{R}_j\hat{R}_i\right\rangle - \left\langle \hat{R}_i\right\rangle\left\langle \hat{R}_j\right\rangle$, $i, j = 1, 2, \ldots, 6$, where $\hat{R} = (\hat{x}_A, \hat{p}_A, \hat{x}_B, \hat{p}_B, \hat{x}_C, \hat{p}_C)^T$ is a vector composed by the amplitude and phase quadratures of tripartite optical beams [2]. Thus covariance matrix of tripartite optical beams can be partially expressed as

*Electronic address: suxl@sxu.edu.cn

(the cross correlations between different quadratures are taken as 0)

$$\begin{aligned}
\sigma_A &= \begin{bmatrix} \triangle^2\hat{x}_A & 0 \\ 0 & \triangle^2\hat{p}_A \end{bmatrix}, \\
\sigma_B &= \begin{bmatrix} \triangle^2\hat{x}_B & 0 \\ 0 & \triangle^2\hat{p}_B \end{bmatrix}, \\
\sigma_C &= \begin{bmatrix} \triangle^2\hat{x}_C & 0 \\ 0 & \triangle^2\hat{p}_C \end{bmatrix}, \\
\sigma_{AB} &= \begin{bmatrix} Cov\left(\hat{x}_A, \hat{x}_B\right) & 0 \\ 0 & Cov\left(\hat{p}_A, \hat{p}_B\right) \end{bmatrix}, \\
\sigma_{AC} &= \begin{bmatrix} Cov\left(\hat{x}_A, \hat{x}_C\right) & 0 \\ 0 & Cov\left(\hat{p}_A, \hat{p}_C\right) \end{bmatrix}, \\
\sigma_{BC} &= \begin{bmatrix} Cov\left(\hat{x}_B, \hat{x}_C\right) & 0 \\ 0 & Cov\left(\hat{p}_B, \hat{p}_C\right) \end{bmatrix}.
\end{aligned} \tag{4}$$

To partially reconstruct all relevant entries of its associated covariance matrix we perform 12 different measurements on the output optical modes. These measurements include the amplitude and phase quadratures of the output optical modes, and the cross correlations $\Delta^2\left(\hat{x}_A - \hat{x}_B\right)$, $\Delta^2\left(\hat{x}_A - \hat{x}_C\right)$, $\Delta^2\left(\hat{x}_B - \hat{x}_C\right)$, $\Delta^2\left(\hat{p}_A + \hat{p}_B\right)$, $\Delta^2\left(\hat{p}_A + \hat{p}_C\right)$ and $\Delta^2\left(\hat{p}_B + \hat{p}_C\right)$. The covariance elements are calculated via the identities [3]

$$\begin{aligned}
Cov\left(\hat{R}_i, \hat{R}_j\right) &= \frac{1}{2}\left[\Delta^2\left(\hat{R}_i + \hat{R}_j\right) - \Delta^2\hat{R}_i - \Delta^2\hat{R}_j\right], \\
Cov\left(\hat{R}_i, \hat{R}_j\right) &= -\frac{1}{2}\left[\Delta^2\left(\hat{R}_i - \hat{R}_j\right) - \Delta^2\hat{R}_i - \Delta^2\hat{R}_j\right].
\end{aligned} \tag{5}$$

In the experiment, we obtain all the covariance matrices of every quantum state actually, and then calculate the PPT value to verify whether the quantum states are entangled or not.

The partially reconstructed covariance matrix of the prepared tripartite entangled optical field is

$$\sigma = \begin{pmatrix} 2.76 & 0 & 2.32 & 0 & 2.27 & 0 \\ 0 & 5.05 & 0 & -2.23 & 0 & -2.27 \\ 2.32 & 0 & 2.78 & 0 & 2.29 & 0 \\ 0 & -2.23 & 0 & 4.81 & 0 & -2.14 \\ 2.27 & 0 & 2.29 & 0 & 2.69 & 0 \\ 0 & -2.27 & 0 & -2.14 & 0 & 4.80 \end{pmatrix}. \tag{6}$$

The entanglement among the prepared tripartite state is evaluated by PPT criterion and we have PPT values $\mathrm{PPT_A} = 0.48$, $\mathrm{PPT_B} = 0.47$, $\mathrm{PPT_C} = 0.48$, respectively.

For the tripartite state distributed over lossy (noisy) channels in the experiment we have

$$\begin{aligned}
\sigma'_A &= \eta_A\sigma_A + (1-\eta_A)\left(g_a N + 1\right) I, \\
\sigma'_B &= \sigma_B, \\
\sigma'_C &= \eta_C\sigma_C + (1-\eta_C) I, \\
\sigma'_{AB} &= \sqrt{\eta_A}\sigma_{AB}, \\
\sigma'_{AC} &= \sqrt{\eta_A\eta_C}\sigma_{AC}, \\
\sigma'_{BC} &= \sqrt{\eta_C}\sigma_{BC},
\end{aligned} \tag{7}$$

where η_A and η_C are the transmission efficiencies of optical modes $\hat{A}$ and $\hat{C}$ respectively, N and g_a represent the excess Gaussian noise in the channel and the magnitude of noise respectively. Different combinations of the parameters correspond to different transmission scenarios. When $\eta_A \neq 1$, $\eta_C \neq 1$ and $g_a N = 0$, the optical modes $\hat{A}$ and $\hat{C}$ are distributed over two lossy channels, respectively, which corresponds to the scenario shown in Fig. 1(a). If $\eta_A \neq 1$, $\eta_C = 1$ and $g_a N = 0$, only mode $\hat{A}$ is distributed over a lossy channel, while modes $\hat{B}$ and $\hat{C}$ are placed within a node without any channel between them. When the optical mode $\hat{A}$ is distributed over a noisy channel, the scenario is shown in Fig. 1(b). In this case we have $\eta_A \neq 1$, $\eta_C = 1$ and $g_a N \neq 0$. After the entanglement revival operation, the elements of the covariance matrix for the output state are

$$\begin{aligned}
\sigma''_A &= \eta_A T\sigma_A + (1-\eta_A T) I, \\
\sigma''_B &= \sigma_B, \\
\sigma''_C &= \sigma_C, \\
\sigma''_{AB} &= \sqrt{\eta_A T}\sigma_{AB}, \\
\sigma''_{AC} &= \sqrt{\eta_A T}\sigma_{AC}, \\
\sigma''_{BC} &= \sigma_{BC},
\end{aligned} \tag{8}$$

respectively, where T is the transmittance of the revival beam-splitter.

[1] van Loock, P. & Braunstein, S. L. *Phys. Rev. Lett.* **84,** 3482 (2000).

[2] Adesso, G. & Illuminati, F. *J. Phys. A: Math. Theor.* **40,** 7821 (2007).

[3] Steinlechner, S., Bauchrowitz, J., Eberle, T. & Schnabel, R. *Phys. Rev. A* **87,** 022104 (2013).
